# Lavandula Species, Their Bioactive Phytochemicals, and Their Biosynthetic Regulation

**DOI:** 10.3390/ijms24108831

**Published:** 2023-05-16

**Authors:** Miroslav Habán, Joanna Korczyk-Szabó, Simona Čerteková, Katarína Ražná

**Affiliations:** 1 Institute of Agronomic Sciences, Faculty of Agrobiology and Food Resources, Slovak University of Agriculture in Nitra, Tr. A. Hlinku 2, 94976 Nitra, Slovakia; miroslav.haban@uniag.sk; 2 Department of Pharmacognosy and Botany, Faculty of Pharmacy, Comenius University in Bratislava, Odbojárov 10, 83232 Bratislava, Slovakia; 3 Institute of Plant and Environmental Sciences, Faculty of Agrobiology and Food Resources, Slovak University of Agriculture in Nitra, Tr. A. Hlinku 2, 94976 Nitra, Slovakia; xcertekova@uniag.sk (S.Č.); katarina.razna@uniag.sk (K.R.)

**Keywords:** lavender, genetic diversity, distribution, secondary metabolites, genetic regulation, microRNAs

## Abstract

Lavandula species are one of the most useful aromatic and medicinal plants and have great economic potential. The phytopharmaceutical contribution of the secondary metabolites of the species is unquestionable. Most recent studies have been focusing on the elucidation of the genetic background of secondary metabolite production in lavender species. Therefore, knowledge of not only genetic but especially epigenetic mechanisms for the regulation of secondary metabolites is necessary for the modification of those biosynthesis processes and the understanding of genotypic differences in the content and compositional variability of these products. The review discusses the genetic diversity of Lavandula species in relation to the geographic area, occurrence, and morphogenetic factors. The role of microRNAs in secondary-metabolites biosynthesis is described.

## 1. Introduction

Lavenders are commonly used as ornamental and melliferous plants [1]. Their essential-oil (EO) content makes them valuable herbs and spices, and of significant importance in industries such as pharmaceuticals, food, flavour, cosmetics, perfumery, and aromatherapy [2]. The pleasant fragrance of lavender is mainly attributed to the content of monoterpenoids, concentrated mainly in the flowers and other upper areas of the plant [3]. The lavender oils that are most valued by the perfumery and cosmetics sectors contain higher levels of linalyl acetate and linalool, but small levels of camphor. On the other hand, those with a higher camphor content are typically used in aromatherapy and phytotherapy [4]. The economic value of *Lavandula* spp. is mainly attributed to the properties of its essential oils, which are subject to strict regulation by international ISO (International Organization for Standardization) standards. These oils have been utilized for cosmetic and therapeutic purposes for centuries, with evidence of their therapeutic use dating back to ancient Greek and Roman times. Recently, the rise in popularity of alternative medicines has sparked a renewed interest in *Lavandula* spp. and their essential oils as natural remedies [5,6]. Lavender essential oils are primarily composed of monoterpenes, with approximately 50–60 monoterpenes having been identified thus far. However, each species has a unique profile in which a select few molecules play a prominent role in determining the aroma and essential-oil properties [6,7]. 

The prevalent lavender varieties are rich in linalool and linalyl acetate, which are the most frequent monoterpenes they contain [8]. The composition of essential oils is primarily influenced by the genetic makeup of the plant, while environmental and propagation factors can also exert a considerable influence. Furthermore, variations in composition may exist among different plant tissues [7,9]. Lavandula species exhibit potential for diverse biological applications, particularly owing to their antioxidant activity. The development of drugs could facilitate the identification and characterization of different bioactive compounds. Investigating the synergistic effects of lavender’s bioactive components with other molecules through in vitro and in vivo studies demonstrates an effective option for the treatment conditions related to impaired immune functions and oxidant stresses. The major constituents of lavender, including volatile oils such as linalool, limonene, perillyl alcohol, linalyl acetate, cis-smine, terpene, coumarin, tannin, caffeic acid, and camphor, vary in relative concentrations among different species [10]. The review provides structured insight into the chemical compositions of lavender species and essential oils. The characteristic constituents and chemotypes are presented. The intra- and interspecific variability is considered with respect to geography, occurrence, and morphogenetic determinants, and genetic diversity. We also summarize the status of genomic and transcriptomic sequencing data, including the genetic background and the role of microRNAs in the secondary-metabolites biosynthesis of lavender species. 

## 2. Lavender Genetic Diversity

*Lavandula* L., a genus of plants, can be found wild in the Mediterranean region from the North Atlantic to the Middle East. These herbaceous biennial plants thrive in dry, sunny environments and grow in either calcareous or siliceous soils depending on the species. The *Lamiaceae* family, to which *Lavandula* L. belongs, is typically defined by the presence of quadrangular stems and opposite, decussate leaves [1,11,12]. 

The Lavandula section comprises compact shrubs that typically reach a height of 20–60 cm and feature wooden stems. The leaves of these shrubs are elongated, in-line, with opposed arrangement. Foliage shape and vanning may differ between lavender species [1,13].

The lavender flowers possess bilabiate form and bee-attracting nectary and are hermaphroditic in nature. Glandular trichomes can be found on the aerial parts of the plant [14,15], and abundance may depend on the species or the specific plant part. Essential oils are contained in this epidermal structure and their form, location, and densities are unique characteristics that distinguish them between the species [14,16,17]. The quantity, abundance, and sizes of glandular trichomes in lavender plants have an impact on the essential oil that is extracted from them [18]. Additionally, different glycosides with taxonomic significance are also present in this family [19].

The family *Lamiaceae* is categorized into seven subfamilies, among which *Nepetoideae* is a subfamily that comprises lavender species. The genus *Lavandula* has been meticulously classified by Upson and Andrews in terms of its taxonomy [1]. The genus *Lavandula* comprises more than 39 species and 79 intraspecific taxa and hybrids, which can be categorized into three subgenera, namely *Fabricia*, *Sabaudia*, and *Lavandula*, as well as eight sections, including *Dentatae*, *Stoechas*, *Lavandula*, *Pterostoechas*, *Subnudae*, *Chaetostachys*, *Hasikenses*, and *Sabaudia* [20]. The most important cultivated species include fine lavender (*L. angustifolia* Mill), lavender aspic (*L. latifolia*), woolly lavender (*L. lanata*), and lavandin (*L. × intermedia*, a sterile hybrid of *L. angustifolia* and *L. latifolia*), and all belong to the Lavandula section. In addition, the genus comprises numerous subspecies and several hundreds of varieties [20,21,22,23]. For essential oil production, the most commonly grown genotypes are *L. angustifolia* and *L. × intermedia* [24,25,26]. Lavender oil (from *L. angustifolia*) is sold at about 3–5× the price of lavandin oil. This is because it is regarded as being of a better standard [27]. The *Lavandula* genus is characterized by a rich content of phenolic compounds, with the identification of eight anthocyanins and nineteen flavones. Additionally, the content of essential oil of *Lavandula* species varies greatly, with over 300 terpenes, including both mono- and sesquiterpenes, having been accounted for [28].

## 3. Lavender Distribution and Occurrence

The distribution of the *Lavandula* genus encompasses a wide geographical range, spanning across the Mediterranean to India, and includes a total of 51 species and hybrids [29,30,31]. In the Mediterranean region, where *Lavandula* species are mainly distributed, 30 species and hybrids can be found in the wild. Among them, 22 species are endemic to the Western Mediterranean, which is considered a centre of diversity for the genus [29,32]. *L. angustifolia*, also known as English or true lavender, is used for gastronomic applications in the Mediterranean. Despite its common name, the native area of this species is restricted to Spain, France, and Italy [29]. 

**Figure 1:**
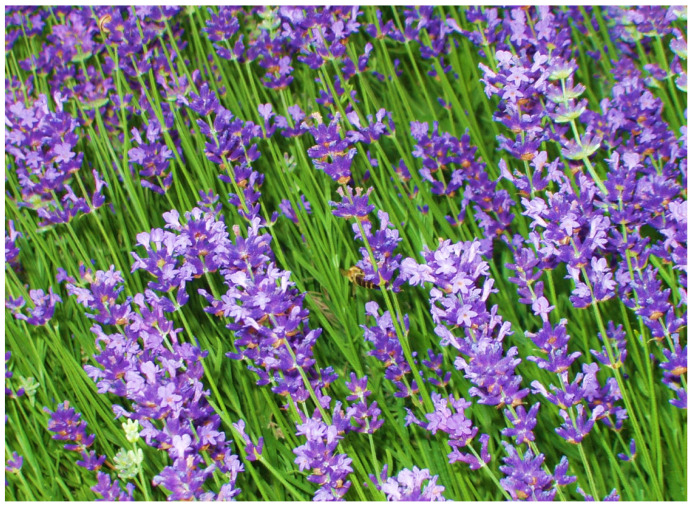
A representative image of lavender from the locality Šaľa (Habán).

**Figure 2:**
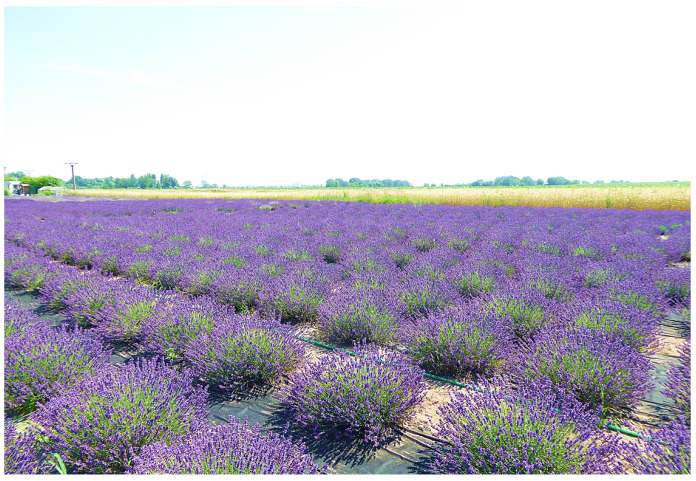
The first Slovak organic lavender farm was established in 2014 in the village of Malé Leváre (Habán).

The cultivation of lavender has a long history in Mediterranean countries such as Italy and France (as shown in Table 1). Some of the localities in Slovakia are presented in Figure 1 and Figure 2). However, in recent times, other countries such as the USA, Canada, Japan, Australia, and New Zealand have emerged as significant commercial producers of lavender [32].

## 4. Lavender Bioactive Phytochemicals and Their Effects

The *Lamiaceae* family is of great economic importance in the botanical world and includes numerous herbs and shrubs that are widely cultivated and utilized for their medicinal or culinary properties throughout the world [14,41,42,43]. Numerous cultivars belonging to the *Lavandula* L. genus have been developed with the primary aim of producing essential oils. Among these, the ‘Maillette’ cultivar is recognized as the standard for high-quality essential-oil production in France. Nevertheless, a handful of other cultivars, such as ‘Compacta’, ‘Irene Doyle’, and ‘Twickel Purple’, exhibit similar desirable characteristics [1]. Certain cultivars of *Lavandula* L. have been identified as particularly suitable for specific applications. For instance, ‘Buena Vista’ has been recommended for potpourri, while ‘Blue Mountain’ is well-suited for use as hedges. Other cultivars have been developed for their ornamental value and exhibit distinctive aesthetic features, including white flowers, light pink hues, lively violet, or dark flowers [24]. The Lavender plant offers a broad spectrum of colours to inspire horticulturists in their selection. Moreover, there are indications that some cultivars are better suited to particular environmental factors, including the ability to survive cold winters [44].

*L. angustifolia* is preferred in the perfumery and cosmetic industries due to its superior volatile profile, whereas other *Lavandula* species with higher volatile compounds that are rich in camphor are utilized in nonaromatic applications [5,45]. Selecting the appropriate cultivar for the intended product is crucial for lavender farmers to maximize their revenue from raw-product processing. Nonetheless, according to the published literature, producers might explore alternative uses for their crops to generate additional income or transition to more profitable applications as well. However, conventional methods for lavender-crop utilization, such as essential-oil extraction, continue to dominate the markets because of strong levels of consumer interest [16].

In the culinary and processing industries, lavender flowers or essential oils may be served as flavouring substances. Their antiseptic aspects may be harnessed to inhibit food deterioration or be added to packaging materials. Lavender essential oil has been examined for its effectiveness in preserving meat, as well as against a broad range of foodborne pathogens [46,47]. The phenolic compounds obtained from the solid residue generated as a byproduct of essential-oil distillation may have various applications in the food sector on account of their antibacterial and antioxidant characteristics. The uses may extend from nutritional additives to package materials [48]. Byproducts of lavender processing that are enriched in polyphenols might be utilized in the bakery sector. For instance, loaves with 2.5% lavender coproduct exhibited increased bread bulk, improved shelf life, and greater consumer acceptability [49]. Lavender essential oil could also be incorporated into active food packaging [50].

The demand for natural ingredients in cosmetics is increasing as consumers seek a healthier lifestyle [51]. Aromatic lavender oil and blooms are used in various applications, including aromatherapy, cleaning products, therapeutic oils, fragrances, and body hygiene preparations [52,53]. Essential oil is applied in small quantities by inhalation or to the body in aromatherapy [46]. The plant material obtained as a coproduct of the extraction of ethereal oils could serve as a source of polysaccharides for use in skin-care preparations [54]. The steam extraction process produces hydrolats as a coproduct in mL amounts that exhibit low antimicrobial activity [55]. It contains other constituents such as linalool, α-terpineol, lavandulol, lavandulyl acetate and caryophyllene, and camphor, and presents a floral–herbal scent [56,57]. The potential uses of lavender hydrolat have been insufficiently explored, and novel applications could be very interesting to the processing industry.

Lavender has a long history of traditional medicinal uses, such as treating wounds, lice, migraines, panic attacks, heart disease, colds, bites, cramps, and congestion, and as a sleeping aid due to the calming effects of lavender teas and extracts [10,46,58]. Some of these traditional uses have been scientifically validated with varying degrees of confidence [5,59]. Studies have found strong evidence supporting the use of lavender in pharmacotherapy for anxiety [11], and moderate evidence for its use as a sedative inducer, for mitigating postnatal pain after childbirth, spasmolytic and antibacterial therapy, and for cancer treatments based on phase I human trials of the lavender constituent perillyl alcohol [10,52]. In vitro studies have also shown cytotoxic effects of specific lavender compounds or essential oils on various cancer cell lines, including breast cancer, leukemia, melanoma, and colon and ovarian cancer cells [6,43].

In addition to its effects on the central nervous system, lavender has been found to possess antispasmodic properties on uterine and intestinal smooth muscle, as well as ergogenic effects in sports training [5] and benefits for the circulatory system [60]. Scientific evidence supports the antioxidant and anti-inflammatory effects of lavender essential oil [51,61,62]. Antimicrobial activity was documented for some common pathogens, such as *Staphylococcus aureus* Rosenbach, *Streptococcus pyogenes* Rosenbach, *Escherichia coli* Mig., *Enterococcus faecalis* (Andrewes and Horder) Schleifer and Kilpper-Bälz [5,11,63,64], *Candida albicans* (C.-P. Robin) Berkhout, *Pseudomonas aeruginosa* (Schröter) Migula, *Bacillus subtilis* (Ehrenberg) Cohn, *Listeria monocytogenes* [18], Pirie, *Salmonella* sp., and *Enterobacter* sp., *Klebsiella* sp. The findings suggest that lavender oil has potential as a preventive agent or local treatment for superficial infections but not for deep infections [55]. However, despite the promising biological activities of lavender, there are several obstacles hindering the development of effective therapies. One major challenge is the absence of standardization in doses and testing protocols across scientific literature, leading to difficulties in achieving a consensus on optimal concentrations [16].

In the field of pharmaceuticals, essential oils are incorporated into various dosage forms, including capsules, creams, syrups, suppressants, and sprays. Lavender essential oil has potential as a medical device and surface sanitizer or as an aerosol in patient operation rooms to limit air infection [46]. Furthermore, by utilizing microorganisms (particularly filamentous fungi) through fermentative or enzymatic processes, the fixed byproduct fractions resulting from essential-oil extraction could be utilized to produce various bioactive compounds with antimicrobial, antioxidant, cosmetic, or pharmaceutical activities. These developments open up new possibilities for applications in white biotechnology [65,66].

## 5. Biosynthesis Regulation of Lavender Bioactive Phytochemicals

**Figure 3:**
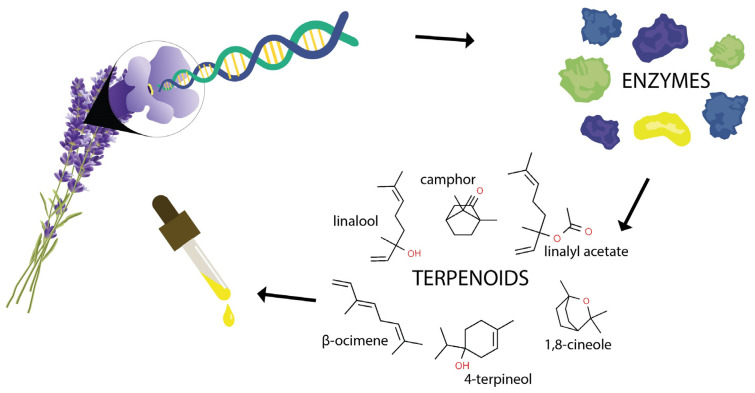
Understanding the complex gene–terpenoid networks is important in the context of improving the phytochemistry of *Lavandula* species through targeted breeding of new varieties (Čerteková).

Many studies have been focusing on the elucidation of the genetic background of secondary metabolite production in lavender species [7,8,67,68]. The most abundant but also the most important secondary metabolites present in lavender essential oil are terpenoids [24,61,69]. Understanding the complex gene–terpenoid networks is important in the context of improving the phytochemistry of *Lavandula* species through targeted breeding of new varieties (Figure 3).

### 5.1. Biosynthesis of Secondary Metabolites in Lavender

Most of the metabolites present in essential oil originate from 5-carbon precursors: isopentenyl diphosphate (IPP) and its isomer dimethylallyl diphosphate (DMAPP). These compounds are synthesized in either the 2-C-methyl-D-erythritol-4-phosphate pathway (MEP) or the mevalonate pathway (MVA). MEP is localized in plastids and generates IPP and DMAPP, which later serve for producing monoterpenes, diterpenes, and tetraterpenes. The first enzyme of this pathway is deoxy-D-xylulose 5-phosphate synthase (DXS). The MVA metabolic pathway takes place in cytosol and peroxisomes. Its products are further involved in the synthesis of sesquiterpenes and triterpenes. The first step of the MVA pathway is catalyzed by the enzyme 3-hydroxy-3-methylglutaryl-CoA synthase (HMGS) and the second by the enzyme 3-hydroxy-3-methylglutaryl-CoA reductase (HMGR) [8,70,71,72]. While the *DXS* gene was highly expressed in the glandular trichomes of the flowers of *L. angustifolia*, the transcripts of the *HMGR* were scarcely detectable. This may indicate the importance of the MEP pathway in the process of secondary-metabolite production [8]. This hypothesis is also supported by another study in which an increased activity was observed in most of the genes involved in the MEP pathway during the flowering period of *L. angustifolia*, whereas genes involved in the MVA pathway were expressed in a smaller quantity [73]. Several sources, however, point to a mutual connection between MVA and MEP pathways [74,75].

IPP and DMAPP are, in the following step, condensed by prenyltransferases to form geranyl diphosphate (GPP) and farnesyl diphosphate (FPP). These molecules can then be used to synthesize a wide variety of terpenoids through the action of TPSs. Hydrolysis of carbocation intermediates derived from GPP produces monoterpenoids, while hydrolysis of FPP produces sesquiterpenoids [75,76]. There are several types of TPSs that significantly contribute to the structural diversity within this class of terpenoids. These enzymes remove the pyrophosphate from linear structures such as GPP and FPP, thereby creating many cyclic or linear products, which can subsequently be subjected to further modifications by hydroxylases, reductases, or cytochrome P450 (CYP) oxygenases [77,78,79,80].

### 5.2. Gene-Based Regulation of Lamiaceae Species Secondary Metabolites

Plants are highly diverse in their chemical and metabolic profiles. In the case of lavender and other species in the *Lamiaceae* family, they have evolved to produce large amounts of terpenoid compounds. This diversity is a result of their adaptation to different environmental conditions throughout the course of evolution and was enabled mainly via the subfunctionalization or neofunctionalization of genes previously duplicated in the events such as small-scale gene duplication (e.g., tandem duplication) or whole genome duplication (WGD) [81]. Enzyme promiscuity (the ability of enzymes to catalyze multiple reactions) and changes in substrate specificity play a role in the metabolic divergence seen in plants [82].

Nonhomologous genes, which encode enzymes involved in the same metabolic pathway are often physically grouped in certain regions of the genome in the form of biosynthetic gene clusters. This enables them to follow similar expression patterns and facilitates coinheritance. The dynamic evolution of genes within these clusters leads to an expansion of the functional diversity of their products [83]. The mechanisms behind the formation of these clusters are not fully understood, but one possible explanation is through the functional divergence of local duplications [84]. Such metabolic blocks were also characterized for genes involved in terpenoid biosynthesis [85,86,87]. In lavender, clusters of *TPS*-*TPS* (terpene synthases), *TPS*-*BAHD*, and *TPS*-*CYP450* were observed. Overall, 34 out of 1181 metabolic clusters identified in lavender were potentially associated with terpenoid biosynthesis [68].

Phylotranscriptomic analyses revealed 28 putative WGDs across the *Lamiaceae* species, with 18 of these WGDs inferred to have occurred within the *Nepetoideae* subfamily. This correlates with the high number of species within this clade [88]. Analysis of the lavender genome revealed that after the core γ triplication, shared among all eudicots, two additional lineage-specific WGD events occurred. In the penultimate WGD event, which occurred ~29.6 million years ago (MYA), 23.1% of the duplicated genes have been retained, while the last WGD event, dated to ~6.86 MYA, retained 32.5% of duplicated genes. The Kyoto Encyclopedia of Genes and Genomes (KEGG) pathway-enrichment analysis revealed that a number of these genes were linked to terpenoid metabolism and plant defence, indicating the importance of terpenoid metabolism in lavender [68].

Poor understanding of the genomic and transcriptomic traits underlying secondary metabolite production in lavender was caused by limited genomic data resources of lavender in comparison to other *Lamiaceae* species. The first genes involved in terpenoid biosynthesis in lavender were identified using a homology-based PCR approach [89]. These genes encode for three terpene synthases (TPSs): (R)-limonene synthase (LaLIMS), (R)-linalool synthase (LaLINS), and trans-alpha-bergamotene synthase (LaBERS), and similar to their homologues [90], each of these genes is composed of six introns with varying lengths [89]. The first expressed sequence tag (EST) library of *L. angustifolia* revealed numerous genes involved in the MVA or MEP pathway as well as genes for known prenyl transferases and TPSs (Lane et al., 2010) [8]. Analysis of EST libraries then became a common approach for identifying new *TPS* genes [7,8,91,92,93]. Many genes associated with terpenoid biosynthesis in lavender species were reported and identified, using the above-mentioned approaches or their combination, which enabled their cloning and further characterization, e.g., 1,8-cineole synthases (*CINS*), borneol dehydrogenase (*BDH*), 9-epi-caryophyllene synthase (*CPS*), lavandulyl diphosphate synthase (*LPPS*), alcohol acetyltransferase-3/-4 (*AAT-3/4*), 3-carene synthase (*3CARS*), and bornyl diphosphate synthase (*BPPS*) were identified in *L.* × *intermedia* [91,92,93,94,95,96,97]; limonene synthase (*LIMS*), linalool synthase (*LINS*), trans-alpha-bergamotene synthase (*BERS*), bornyl diphosphate synthase (*BPPS*), β-phellandrene synthase (*βPHLS*), and acyltransferase-1/2 (*AT1/2*) were identified in *L. angustifolia* [89,91,98,99] and fenchol synthase (*FENS*), α-pinene synthase (*PINS*), and germacrene A synthase (*GEAS*) in *L. pedunculata* Mill. [100]. The EST technique, however, has proven to be insufficient in identifying nonabundant transcripts and in recent years has been replaced by more comprehensive and cost-effective sequencing methods that allow for the analysis of the entire transcriptome, rather than just a small portion, and provide more detailed and accurate information about gene expression levels and patterns [73,97,101].

Genome and transcriptome sequencing data provide unprecedented insight into the genetic basis of lavender. Published in 2018, the first draft genome of *L. angustifolia* (cv. Maillette), with a total length of 870 Mbp [67], revealed 62,141 protein-coding genes and 2003 noncoding genes (tRNA/rRNA). The latter reported the chromosome-level genome of *L. angustifolia* (cv. Jingxun 2), consisting of 894.5 Mbp anchored to 27 chromosomes. This genome assembly revealed 65,905 protein-coding sequences, of which 62,822 (95.3%) had annotated homologs in the database. The number of noncoding RNAs was estimated to be 1351 miRNAs, 1298 tRNAs, 1199 snRNAs, and 399 rRNAs [68]. Both genome assemblies have a GC content of approximately 38%. A significant difference was observed between the assemblies of the two cultivars in the density of repetitive sequences. In the case of the Maillette cultivar, it was estimated that 43% of the genome consists of repetitive sequences, while a higher proportion of 58% was reported for the Jingxun 2 cultivar. Similar to other species, LTR retrotransposons are the most prevalent type of repeated sequences in lavender [67,68]. Evaluation using benchmarking universal single-copy orthologues (BUSCO) indicated a high degree of completeness of both published assemblies.

The first draft genome revealed sequences for all known genes involved in MEP and MVA pathways, most of them present in more copies. Genes for the enzymes catalyzing the first and last step of the MEP pathway, *DXS*, and *HDR*, were present in 13 and 7 copies, respectively. When compared to genomes of *Mentha longifolia* (L.) Huds., *Arabidopsis thaliana* (L.) Heynh., *Zea mays* (L.), and *Oryza sativa* (L.), the highest copy number of the genes involved in terpenoid biosynthesis was observed in lavender [67]. A high copy number of these genes in the genome of lavender demonstrates a high degree of optimization for EO production.

*TPS* genes can be grouped into seven subfamilies [102]. In lavender, five of these subfamilies were found to be present and, overall, 100 genes for TPS were identified. The *TPS-b* subfamily, which typically encodes monoterpene synthases, was the most expanded one in lavender and genes belonging to this subfamily showed high levels of expression in the glandular trichomes of *L. angustifolia* [67].

### 5.3. The Role of Transcription Factors and microRNAs in the Biosynthesis of Secondary Metabolites

Gene regulation provided by transcription factors (TFs) is crucial for ensuring that the correct genes are activated or repressed at the appropriate time and place, thereby allowing cells to respond to different stimuli and perform diverse functions. Study which examined TFs that regulate the expression of key genes involved in monoterpenoid biosynthesis in *L. × intermedia* indicated that the *LINS* and *CINS* genes are regulated by TFs that possess conserved domains that are typical of MYB, bZIP, NAC, GeBP, and SBP-related proteins [103]. The results of the study indicate that the *LINS* and *CINS* genes are regulated by TFs that possess conserved domains that are typical of MYB, bZIP, NAC, GeBP, and SBP-related proteins.

Structural genes, and also TFs themselves, can be regulated by small noncoding RNA molecules—miRNAs. MiRNAs regulate gene expression by binding to complementary sequences in messenger RNA, leading to the degradation or repression of translation. A considerable amount of literature has been published on the regulatory role that miRNAs play in the biosynthesis of secondary metabolites [104,105,106]. Computational analysis of miRNA molecules involved in secondary metabolism in *Mentha* spp. revealed 11 miRNA families, which targeted 130 transcripts involved in various metabolic processes. The biosynthesis of terpenoids is inferred to be regulated by miR156, miR414, and miR5021, while trichome development is associated with miR156, miR5021, and miR5015b. Out of all identified miRNAs, miR5021 regulated the largest number of transcripts involved in the secondary metabolism of *Mentha* spp. [104].

In other model plants, it was also reported that miR5021 is involved in terpenoid backbone biosynthesis by targeting *HMGR*, isopenteyl diphosphate isomerase (*IDI*), hydroxymethylbutenyl diphosphate synthase (*HDS*), geranylgeranyl diphosphate synthase (*GGDPS*), diphosphomevalonate decarboxylase (*MVD*) [107,108,109], and transcription factors belonging to MYB family, which, among other things, were proven to be involved in the regulation of sesquiterpene and diterpene biosynthesis [107,110,111]. In *Ocimum basilicum* L., miR5021 may also play an important role in cysteine and methionine metabolism [112]. The regulation role of miR156 on terpenoid biosynthesis is likely to be mediated by the squamosa promoter binding protein-like (SPL) transcription factor. It has been reported that miR156 targets the SPL transcription factor in both *A. thaliana* (L.) Heynh and patchouli (*Pogostemon cablin* Benth), which is also a member of the *Lamiaceae* family. By binding to its promoters, SPL activates genes of sesquiterpene synthases (*TPS21* in *A. thaliana* and PTS in patchouli), thus increasing the production of sesquiterpenes [113].

Analysis of small RNA (sRNA) libraries obtained via tissue-specific sequencing of *Salvia miltiorrhiza* Bunge revealed 452 known miRNAs and 40 novel miRNAs, all of which could be classified into 62 miRNA families. Tissue-specific expression patterns were observed for numerous miRNAs identified in this study. Using a degradome sequencing approach, followed by gene ontology (GO) and KEGG pathway analysis, it was discovered that miR5072 cleaves acetyl-CoA C-acetyltransferase, which is involved in the biosynthesis of terpenoids [114]. Another study identified 19 potential target genes for 18 miRNAs predicted from the ETS library of *Salvia sclarea* L. In addition to miR156 regulating SPL expression, miR828 was found to target MYB family transcription factors, while AP2 domain-containing transcription factors mRNAs were targeted by miR172 [115]. AP2 domain-containing transcription factors were first linked to terpenoid metabolism in *Catharanthus roseus* (L.) G. Don. [116]. Significant downregulation in sesquiterpenoid and triterpenoid biosynthesis pathways was observed in *S. miltiorrhiza* hairy roots, in which transcription factors belonging to AP2/ERF family were overexpressed [106]. 

A total of seven miRNA families were computationally identified in *O. basilicum*. Further analysis revealed 13 potential targets for four of these miRNA families, with the majority of these targets being stress responsive [112]. The number of studies focusing on possible miRNA cross-kingdom targets is also on the rise. For example it was found that eight successfully identified miRNA families in the *O. basilicum* genome could potentially regulate up to 87 human genes associated with RAS/MAPK signalling cascade, cardiomyopathy, HIV, breast cancer, lung cancer, Alzheimer’s, and other neurological disorders [117]. The summary of known miRNAs included in the terpenoid biosynthesis is presented in Table 2.

## 6. Conclusions

Diversity in the production of large amounts of terpenoid compounds in the lavender species is a result of their adaptation to different environmental conditions. The benefits of lavender are unquestionable, as evidenced in the review, focusing on the genetic diversity, distribution, abundance, and importance of lavender’s bioactive compounds. We summarised the current knowledge of the genomic and transcriptomic data of traits underlying secondary metabolite production in lavender. Gene regulation by transcription factors is crucial to ensure that the appropriate genes are activated or repressed at the time and place that enables cells to respond to different stimuli and undertake various functions. However, structural genes and transcription factors can be regulated by small noncoding RNA molecules–miRNAs. An area of intense research is the regulatory role of miRNAs in the biosynthesis of secondary metabolites in *Lamiaceae* species. The predominant approach is the computational analysis of miRNA molecules involved in secondary metabolism synthesis. In terms of the prospective use of lavender species, we highlighted genetic and epigenetic mechanisms regulating the biosynthesis of secondary metabolites, their content, and composition that predetermine the use of lavender.

## Figures and Tables

**Table 1:** Distribution and cultivation of lavender.

Country	Locality	References
France	Provence (Valensole), Lourmarin, Grass, Sisteron	[29]
Italy	Piemont (Demont), Andonno, Civitella Marittima, Borgo, Mozzano	[29,33]
UK	Catswold, Hitchin (Hertfordshire, Bedfordshire), Banstead (Woodmansterne–Surrey Hampshire), Lordington (West Sussex), Heacham, Terrington (York)	[24]
Canada	Vancouver, Campbellville, Windham Centre, Campbellcroft–Port Hope, Niagara on The Lake, Seagrave, Dundas, Waterford, Greenbank, Ayr (Brant)	[32]
USA	Oregon, Matanzas Creek Winery, Mt. Shasta Lavender Farms, Montague (California), Blanco (Texas), Soleado (Maryland), Lavender by the Bay (New York), Sequim, (Washington)	[24]
Australia	Nabowla TAS, Wandin North VIC, Laggan NSW, Mount Alford QLD, Lyndoch SA, Carlotta WA, Pemberton WA	[24]
Japan	Furano, Tomita (Hokkaido)	[24,32]
Bulgaria	Szumen, Dobricz, Karlovo	[34]
Croatia	Hvar (Velo Grablje)	[35]
Poland	Kujawy (Grebocin, Złotniki), Podlasie (Dworzysk, Dzięciołowo), Wielkopolska (Pakszyn, Kicin, Krzemieniewo, Kotuń, Jutrosin, Murzynowo, Ląd, Sławno), Malopolska (Masłomiąca, Ostrów-Kraków), Łózkie (Wieluń, Uniejów, Zelów), Dolnośląskie (Świdnica, Oborniki, Gryfów), Lubelskie (Siedliszcze, Kiełczewice, Końskowola), Lubuskie (Silna), Mazowieckie (Ryczołek, Korabiewice, Borkowice, Borowiczki, Siwianka), Opolskie (Grodków, Zdziechowice, Biadacz), Warmińsko-Mazurskie (Jonkowo), Pomorskie (Rotmanka, Przywidz, Górzyca)	[36,37]
Slovakia	Šaľa (Šaľa), Malé Leváre (Malacky), Modra (Pezinok), Oščadnica (Čadca), Tomášikovo (Galanta), Východná (Liptovský Mikuláš), Branovo (Nové Zámky), Stankovce (Trebišov), Kapoňa–Leles (Trebišov)	[38]
Czechia	Starovičky (Morava), Ostrava-Poruba, Vysočina (Třebič), Bezděkov (Klatovy), Židovice (Litoměřice), Strání (Uherské Hradiště), Chodouň (Beroun)	[39]
Hungary	Tihany (Balaton)	[40]

**Table 2:** miRNA involved in the terpenoid biosynthesis regulation.

miRNA	Target	Plant Species	Reference
miR156	deoxy-D-xylulose 5-phosphate synthase SPL transcription factor	*Mentha* spp. *Salvia sclarea* L.	[104] [115]
miR414	terpene synthase 21	*Mentha* spp.	[104]
miR5021	deoxy-D-xylulose 5-phosphate synthase isopenteyl diphosphate isomerase geranylgeranyl pyrophosphate synthase	*Mentha* spp.	[104]
miR5072	acetyl-CoA *C*-acetyl transferase	*Salvia sclarea* L. *Salvia miltiorrhiza* Bunge	[115] [114]
miR172	AP2 domain-containing transcription factor	*Salvia sclarea* L.	[115]
miR828	MYB12	*Salvia sclarea* L.	[115]
miR858	MYB family transcription factor	*Salvia miltiorrhiza* Bunge	[114]

## Data Availability

Data sharing not applicable.
